# Whole-Genome Sequence and Methylome Analysis of the Freshwater Colorless Sulfur Bacterium *Thioflexothrix psekupsii* D3

**DOI:** 10.1128/genomeA.00904-17

**Published:** 2017-08-31

**Authors:** Alexey Fomenkov, Tamas Vincze, Margarita Y. Grabovich, Galina Dubinina, Maria Orlova, Elena Belousova, Richard J. Roberts

**Affiliations:** a New England Biolabs, Ipswich, Massachusetts, USA; b Department of Biochemistry and Cell Physiology, Voronezh State University, Voronezh, Russia; c Federal Research Center for Fundamentals of Biotechnology, Russian Academy of Sciences, Moscow, Russia

## Abstract

In this report, we announce the availability of a whole-genome sequence and methylome analysis of *Thioflexothrix psekupsii* strain D3.

## GENOME ANNOUNCEMENT

At the end of the 19th century, Sergei Winogradsky introduced the concept of chemolithotrophy when he first reported on organisms gaining energy exclusively from the oxidation of inorganic compounds (1). Members of the bacterial family *Beggiatoaceae* have gained much attention due to their ability to oxidize sulfide to elemental sulfur, which they deposit intracellularly in the form of small globules or droplets. However, due to the difficulties of isolation, purification, and growth of this bacterium *ex situ*, only a few draft genome sequences have been assembled (GCA_000170695.1; GCA_000170715.1). 

Among the many morphotypes of the *Beggiatoa* genus that have been described in the literature, only two species, *B. alba* (NCBI reference number NZ_AHMA00000000) and *B. leptomitoformis* D401 and D402, have been subjected to genome sequencing (CP018889 and CP012373) (2). Here, we announce the availability of a whole-genome sequence and methylome analysis of a new member of the *Beggiatoaceae* family, *Thioflexothrix psekupsii* D3. This strain has been described previously based on its morphological and biochemical characteristics (3). *T. psekupsii* D3 produces immense amounts of exopolysaccharides (EPSs), mostly consisting of galactose polymers, during growth. The ratio of synthesized EPSs to the cellular proteins is about 10 to 1 (4). Therefore, isolation and purification of high-quality, high-molecular-weight DNA from this bacterium was quite a challenge. Several attempts to purify the DNA from 1 liter of growth culture resulted in very limited amounts, between 300 and 1,000 ng, with a fragment size range of 2 to 5 kb. We separated residual EPSs from DNA by purification on PowerClean columns (Mo Bio Laboratories, Inc., Carlsbad, CA, USA), prepared libraries using PreCR, and performed end repair and ligation to hairpin adapters using a PacBio protocol adapted for NEB components. Genomic DNA fragment and SMRTbell library qualification and quantification were performed using the Qubit fluorimeter (Invitrogen, Eugene, OR, USA) and the 2100 Bioanalyzer (Agilent, Santa Clara, CA, USA). Two SMRTbell libraries of 2 and 5 kb were sequenced using C2-P4 and C4-P6 chemistry on eight and two single-molecule real-time (SMRT) cells with 180- and 240-min collection time protocols, respectively. Sequencing reads were processed, mapped, and assembled by the Pacific Biosciences SMRT Analysis pipeline using the HGAP3 protocol and polished using Quiver (5). The best assembly from the 5-kb library resulted in a 4,010,614-bp genome size consisting of four major contigs of 3,572,323 bp, 247,009 bp, 62,078 bp, and 16,849 bp with 283× coverage and 21 small contigs (range 4,335 to 5,926 bp) with 20× coverage. The assembled sequences were annotated with the Rapid Annotations using Subsystems Technology (RAST) server (6) and the NCBI Prokaryotic Genome Annotation Pipeline (PGAP) and have been deposited at DDBJ/EMBL/GenBank (MSLT00000000).

Epigenetic modification at each nucleotide position was measured as kinetic variations (KVs) in the nucleotide incorporation rates, and methylated motifs were deduced from the KV data (7–9).

A total of 17 DNA methyltransferase recognition motifs were directly detected by the SMRT motif and modification analysis pipeline. Nine of them contained m6A, and seven contained m4C modifications. Two additional m5C methylase genes were predicted based on homology to known methyltransferase genes. Matching of motifs with methyltransferase genes was carried out, and the results are shown in Table 1. The results have also been deposited in REBASE (10).

**TABLE 1:** Summary of DNA methyltransferases and modified motifs identified in *Thioflexothrix psekupsii* D3

Motif	Assigned MTase	Type of methylation	RM system type^b^
Motif detected^a^			
**A**AGCTT	M.TpsD3I	m6A	II beta
CGARR**A**C	M.TpsD3II	m6A	II G, S gamma
**C**CGG	M.TpsD3III	m4C	II beta
GATC	M.TpsD3IV	m6A	II alpha
GGNC**C**	M.TpsD3V	m4C	II alpha
C**C**WGG	M.TpsD3VI	m4C	II alpha
GG**C**C	2 candidates	m4C	II alpha
ACC**C**C		m4C	III
CCG**A**G		m6A	III
CGARC**A**		m6A	II
CTTC**A**G		m6A	II
DCG**A**GG		m6A	II
GGA**A**YGC		m6A	II
GGANS**A**		m6A	II
GGDH**C**C		m4C	II
Motif predicted from genomic sequence			
RGCGCY	M.TpsD3ORF11940P	m5C	II
GCCGGC	M.TpsD3ORF5P	m5C	II
ATGCAT	M.TpsD3ORF16195P	m6A	II beta

a Modified bases are in bold or underlined if on the complementary strand.

b RM, restriction-modification.

### Accession number(s).

The whole-genome sequence and analysis of the *T. psekupsii* D3 are available in GenBank under the accession number MSLT00000000.
